# Unexpected presentation of accessory breast cancer presenting as a subcutaneous mass at costal ridge: a case report

**DOI:** 10.1186/s13256-020-02366-0

**Published:** 2020-03-31

**Authors:** Peeradech Thasanabanchong, Mawin Vongsaisuwon

**Affiliations:** Department of Surgery, Faculty of Medicine, Chulalongkorn University, Bangkok, Thailand

**Keywords:** Breast cancer, Ectopic breast cancer, Breast, Ectopic breast, Subcutaneous mass

## Abstract

**Background:**

During embryogenesis, bilateral thickening of ectoderm from anterior axillary folds to inguinal folds, called mammary ridges or milk lines, develops into breast tissues. Only a pair in the pectoral area is spared from regression and continuously develops into normal breasts. Accessory breasts can result if the regression process is incomplete. These ectopic breasts can change physiologically and pathologically similar to normal breasts. Unsurprisingly, they are capable of turning malignant. Reported cases show the most common area for accessory breast cancer to be the axillary area. We report a rare case of accessory breast cancer over the costal ridge.

**Case presentation:**

We present the case of a 51-year-old Asian woman who complained of an enlarged mass lower to her left breast developed over the period of 3 months while on contraceptive pills. Unaware that the mass could be an accessory breast, the primary doctor had prescribed oral contraceptives. After our patient had noticed that the mass was obviously growing, she decided to consult a surgeon as the mass continued to grow. Expected to be benign, the mass was investigated by ultrasonography and then excised surgically. A pathology report identified the mass to be a carcinoma of the ectopic breast tissue. However, sentinel lymph node biopsy showed no nodal metastasis of all four lymph nodes. Following surgery, she received chemotherapy, radiation, and antihormonal treatment. After 2 years of postoperative follow up, she remained free of disease.

**Conclusions:**

This patient was diagnosed as having accessory breast cancer which presented with a subcutaneous mass. As this condition is exceptionally rare but curable by early treatments, we recommend surgeons to consider potential malignancy when patients present with a subcutaneous mass.

## Introduction

Breast cancer is one of the most common cancers in women. Of all cases of breast cancer, 0.3–0.6% of cases are ectopic [1]. Although accessory breast cancers (ABCs) mostly occur in the axillary area [2], they can be found in other areas, for example, scapula, thigh, and labia majora [3]. We found an exceptionally rare case of ABC where a subcutaneous mass without any overlying skin changes was located over the costal ridge. ABCs should undergo triple assessment, including clinical examination, radiological imaging, and tissue sampling, as with typical breast cancer [1]. TNM classification should follow the diagnosis of ABC [4], after which wide local excision with or without adjuvant therapies is the standard treatment [1]. This case report describes our management of a patient with ABC.

## Case presentation

A 51-year-old Asian woman presented to a gynecologist with an irregular menstrual cycle, for which she was prescribed oral contraceptives for 3 months. During that period, she complained of a growing, preexisting mass inferior to her left breast over the costal ridge. This painless mass showed no skin hyperpigmentation or visible small blood vessels. She reported occasional congestion a couple of days prior to menstruation as a result of this mass. No oozing secretion was ever detected when breastfeeding. She did not have a fever or experience any changes in appetite or weight at the time of the visit, after which she was referred to a surgeon.

Her weight was 49.40 kg and her height was 150 cm, with a body mass index (BMI) of 21.96 kg/m^2^. She did not have any underlying diseases and was not on any medications but the prescribed oral contraceptives. Her first menstruation came at 14. She currently has two children, giving birth to the first at 32. She breastfed her first child and her second child for 2 years and 1 year, respectively. Prior to the aforementioned treatment for her irregular menstrual cycle, she had not received any contraceptive or hormonal drug. There was no history of alcohol consumption or tobacco smoking. There was no family history of breast and gynecologic cancer. Her maternal aunt was diagnosed as having colon cancer in the eighth decade of life.

At the first physical examination, she had no fever. Her vital signs showed a pulse rate of 80 beats per minute, a respiratory rate of 18 times per minute and a blood pressure reading of 126/72 mmHg. A well-defined round mass of 2 cm diameter was detected inferior to her left breast. This mass was not attached to the skin or chest wall and did not appear to cause any inflammation or skin retraction. No abnormality was detected at the equivalent location on her right breast. Axillary and supraclavicular lymph nodes were also not palpable.

The mass was initially investigated by ultrasonography. Several lobulated hypoechoic nodules were shown outside the breast tissue, inferior to our patient’s left breast: a large nodule of 15.6 × 9.5 mm and two smaller ones of 4.0 × 2.3 and 2.7 × 1.6 mm with marked hypervascularization. Her mammographic finding was classified into Breast Imaging Reporting and Data System (BIRADS) 3, showing mild scattering, round and benign microcalcifications in both breasts, and unremarkable visualized axillary lymphadenopathies.

**Fig. 1:**
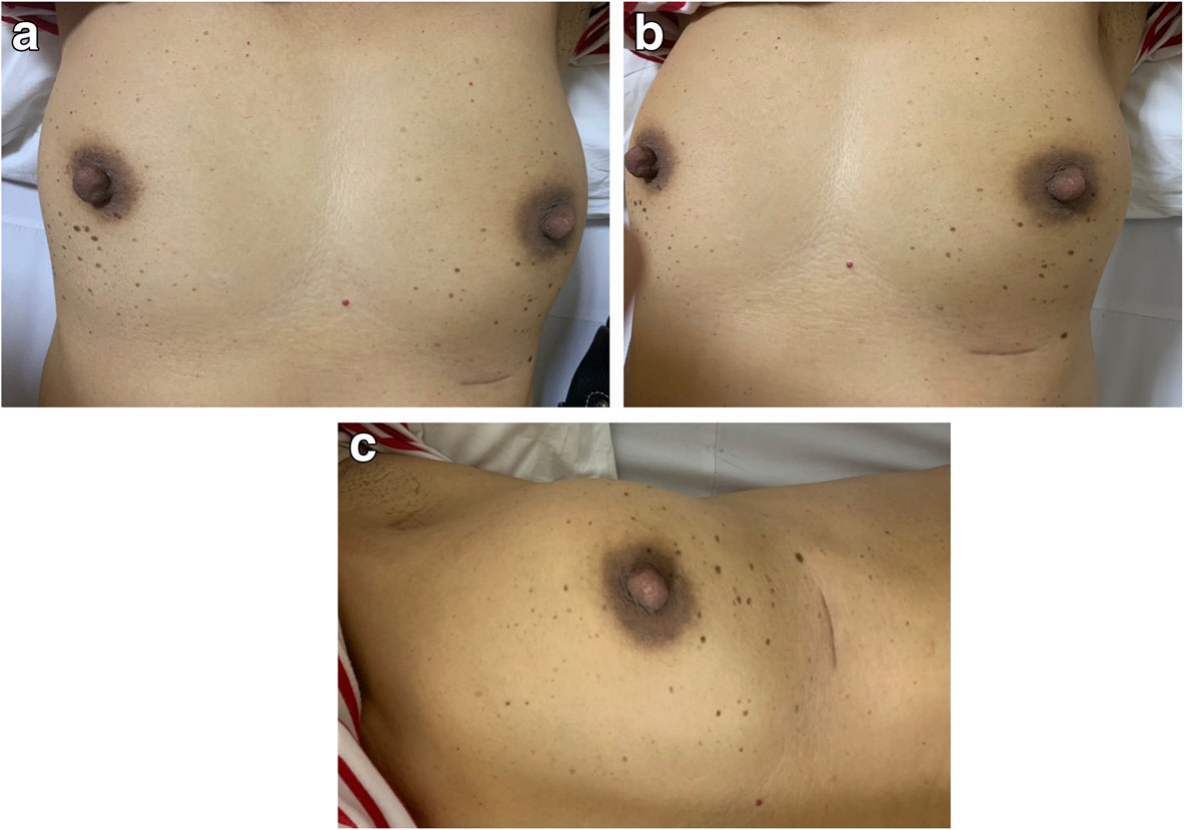
Postoperative wound – this figure shows the site of postoperative wound over the costal ridge inferior to the left breast

The mass was excised (Fig. 1) without suspicion of malignancy and the tissue subjected to pathological examination. A pathology report showed the 2 × 1.8 × 1.5-cm tumor to be a poorly differentiated adenocarcinoma, morphologically consistent with invasive ductal carcinoma of no special type arising in ectopic breast tissue. All margins were negative. No lymphovascular invasion was seen. For immunohistochemical studies, estrogen receptor (ER) was negative but the positivity of the receptors of progesterone (PgR) was 40% of the neoplastic cells. Her2/neu was negative. Ki-67, used as a proliferation index, was 70%. The tissue was considered to be the luminal B subtype.

As the tissue was malignant, nodal metastasis must be considered. Seven days following excision, the surgeon performed sentinel lymph node biopsy in our patient. Radionuclide was injected into the left periareolar area and the lesion site where the excision was done. No metastatic neoplasm presented in any of all four lymph nodes. We then considered this a typical breast cancer with TNM classification of T1cN0M0, stage Ia.

She was scheduled for adjuvant therapy, in which she received four cycles of chemotherapy with cyclophosphamide 960 mg and docetaxel 120 mg per cycle. She then received hypofractionated and boost radiation (42.5 Gy in 16 fractions and boost doses of 10 Gy). Tamoxifen was prescribed as an antihormonal therapy following the radiation. No serious adverse effect resulted. After 2 years of follow up, no evidence of disease recurrence was detected by physical examination and mammography.

## Discussion

Embryogenically, breast tissue develops from the thickening of the ectoderm termed the embryonic mammary ridge, also known as the milk line, extending bilaterally from the anterior axillary folds to the inguinal folds in the sixth week of gestation. These mammary ridges should regress, while those of the pectoral area, where normal breasts are, are spared. If the embryological regression is incomplete, accessory breast tissues develop. Ectopic breast tissues are classified as supernumerary breast tissue and accessory breast tissue [5]. Accessory breast tissue may be divided into polymastia (breast tissue composed of ductal system connecting to overlying skin) and polythelia (accessory nipple or areola presented by a cluster of hair). Aberrant breast tissue is disorganized secretory glandular tissue not connected to skin [3]. Ectopic breast tissues are uncommon in the general population with 0.6–6% occurrence [6]; ectopic breast tissues occur more commonly in women than in men. While the most common site is the axillary area [2], ectopic breast tissues can also be found in the scapula, thigh, and labia majora [3]. These accessory breast tissues may consist of one or any combinations of the nipple, areola, and breast parenchyma [7] and can undergo physiologic changes such as swelling, tenderness, and secretion during puberty, pregnancy, and lactation, as is the case with normal breasts [8]. Accessory breast tissue can also undergo pathologic changes both benign and malignant from fibroadenoma and [9] intraductal papilloma [10] to carcinoma formation. Carcinoma of accessory breast tissues is rare but it is more common in aberrant breast tissue not in the milk line than in supernumerary breasts [5]. In our case, oral contraceptive pills were prescribed to our patient for 3 months without awareness that the growing mass she reported was an accessory breast responding to the prescribed hormones. Diagnosing such cases of breast cancer can in fact be challenging.

Ectopic breast cancer, accountable for 0.3–0.6% of all breast cancers [1], may remain undetected until it changes significantly, for instance, grows in size. Even then, it is often confused with other lesions such as subcutaneous lipoma. As such, microscopic pathology plays a crucial role in confirming the diagnosis. The juxtaposition of normal breast tissue and breast carcinoma tissue must be present histologically to conclude that accessory breast carcinoma is the primary lesion. In addition, lymph node tissue must be absent to exclude the possibility of the lesion being a metastatic tumor [11]. From existing literatures, ABCs were mostly reported in the axillary area. We report a particularly rare case of ABC detected over the costal ridge.

Carcinoma of ectopic breast tissue should be diagnosed by history taking, physical examination, imaging (ultrasonography and mammography), and tissue sampling, such as fine-needle aspiration (FNA) and core-needle biopsy (CNB) [1]. Once it is diagnosed, TNM classification should be considered [4]. Wide local excision of the mass with regional lymph node staging followed by adjuvant therapies, including radiation, chemotherapy, and antihormonal therapy, are considered the best treatment options [1].

Sappey’s line, defined as an imaginary line transversely connecting the umbilicus to the L2 area on the back, separates the area of lymphatic drainage. The lymphatic drainage of the skin and subcutaneous tissue above Sappey’s line is believed to drain into the ipsilateral axillary nodes, while that below Sappey’s line is believed to drain into the ipsilateral groin nodes [12]. This primary concept spearheaded our plan of care for this patient. If the lymph nodes were positive for malignancy, we would have dissected the axillary lymph nodes.

Sentinel node mapping can be used as lymph node staging; however, previous surgery is believed by experts to interfere with lymphatic flows, making sentinel node mapping an unacceptable tool in determining the original lymphatic pathways [13]. In this case, where there was no compelling sign of ABC initially, we did not perform tissue sampling as we thought the mass was a subcutaneous lipoma. If FNA had been done and the result positive for malignancy, sentinel lymph node study would have been performed prior to the excision, when the lymphatic pathways were not yet distorted by surgery and mapping was still possible.

It is suggested that patients who have accessory axillary breasts should be screened for benign and malignant diseases in both their pectoral breasts [3]. Our patient was screened by physical examination and mammography. The prognosis for ectopic breast cancer is poorer than that of breast cancer in the general population, an outcome that may have less to do with the natural disease characteristics and more to do with the rarity of ABC, resulting in less clinical suspicion [4]. Although ectopic breast cancer can make patients more vulnerable to second primary breast cancer, this risk is not strong enough to perform ipsilateral mastectomy for prophylaxis [4]. The prognosis of ectopic breast cancer depends on nodal metastasis, as is the case with typical breast cancer [14].

## Conclusion

This patient presented with a subcutaneous mass and was diagnosed as having ABC, which is rare and usually underestimated. As it was not present with nipple-areolar complex, it may be diagnosed as a lipoma, lymph node, sebaceous cyst, or hidradenitis suppurativa [1]. Conditions related to accessory breasts, especially malignant conditions, should be considered when patients present with a subcutaneous mass located in bilateral milk lines. Due to the aforementioned possibility, tissue sampling of a subcutaneous mass may be advantageous for diagnosis and management plan. ABC must be quickly detected and diagnosed when the disease is still curable. Because ABC appears to have a poorer prognosis than breast cancer of parenchyma [1], timely and proper management should be provided.

## Data Availability

Not applicable.

## Abbreviations

ABC: Accessory breast cancer
FNA: Fine-needle aspiration
